# Long-term outcomes of slipped capital femoral epiphysis treated with in situ pinning

**DOI:** 10.1007/s11832-016-0759-z

**Published:** 2016-07-20

**Authors:** Jolanda J. de Poorter, Tom J. Beunder, Barzi Gareb, Hubert J. Oostenbroek, Gert H. J. M. Bessems, Joris C. T. van der Lugt, Patrick G. M. Maathuis, Michiel A. J. van der Sande

**Affiliations:** 1 Department of Orthopaedics, Erasmus Medical Center, Rotterdam, The Netherlands; 2 Department of Orthopaedics, Leiden University Medical Center, Leiden, The Netherlands; 3 Depatment of Orthopaedics, University of Groningen, University Medical Center Groningen, Groningen, The Netherlands; 4 Department of Orthopaedics, Hagaziekenhuis, The Hague, The Netherlands

**Keywords:** Slipped capital femoral epiphysis, Adolescents, Hip, Osteoarthritis, PROMs

## Abstract

**Purpose:**

Slipped capital femoral epiphysis (SCFE) is the commonest hip disorder in adolescents. In situ pinning is commonly performed, yet lately there has been an increase in procedures with open reduction and internal fixation. These procedures, however, are technically demanding with relatively high complication rates and unknown long-term outcomes. Nevertheless, reports on long-term results of in situ fixation are not equivocal. This study evaluates the possible higher risk of worse outcome after in situ pinning of SCFE.

**Methods:**

All patients treated for SCFE with in situ fixation between 1980 and 2002 in four different hospitals were asked to participate. Patients were divided into three groups, based on severity of the slip. Patients were invited to the outpatient clinic for physical examination and X-rays, and to fill out the questionnaires HOOS, EQ5D, and SF36. ANOVA and chi-squared tests were used to analyze differences between groups.

**Results:**

Sixty-one patients with 78 slips filled out the questionnaires. Patients with severe slips had worse scores on HOOS, EQ5D, and SF36. 75 % of patients with severe slips had severe osteoarthritis, compared to 2 % of mild and 11 % of moderate slips.

**Conclusion:**

Hips with mild and moderate SCFE generally had good functional and radiological outcome at a mean follow-up of 18 years, and for these hips there seems to be no indication for open procedures. However, severe slips have a significantly worse outcome, and open reduction and internal fixation could therefore be considered.

## Introduction

Slipped capital femoral epiphysis (SCFE) is the most common hip disorder in adolescents, with a prevalence of 10.8 cases per 100,000. SCFE mostly occurs in children 9–16 years [1, 2]. Although the etiology of SCFE remains unclear, it has been shown that obesity, male gender and endocrine abnormalities are risk factors for development of the condition [3, 4].

The slipped femoral head displaces to posterior and inferior, thereby creating a varus, extension, and external rotational deformity in the neck of the femur [5]. Once SCFE is diagnosed, semi-urgent treatment is indicated to prevent progression of the slip. For a stable SCFE, in situ fixation is commonly performed. For unstable SCFE, urgent but gentle reduction and internal fixation, with or without decompression, is commonly advocated [4]. The pinning itself is solely intended to stabilize the femoral head, but the possible consequences of a non-anatomical position of the epiphysis remain present. However, long-term follow-up studies have shown that some remodeling occurs and that the loss of internal rotation is not clinically relevant [5]. When remodeling is not sufficient, impingement of the femoral head in the acetabulum can occur with associated development of early onset osteoarthritis of the hip [6]. Data on long-term functional results of in situ pinning are limited. Many of the studies include other treatments than in situ pinning or include older treatment techniques like non-operative treatment or pinning after closed reduction [7, 8].

To improve the postoperative hip dysfunction and malposition of the femoral head, several osteotomies have been described, mostly with satisfactory results [9, 10]. Recently, some authors have focused on initial open reduction and internal fixation of unstable [11] as well as severe but stable [12] slips. These procedures, however, are technically demanding and prone to complications and there are no reports describing long-term outcomes [13].

In our clinical experience, some patients have almost no symptoms or functional problems after in situ pinning. However, another group of patients does have persistent symptoms and develop early osteoarthritis. This study aims to assess what functional problems patients experience after in situ pinning of SCFE and which patients have a higher risk of worse functional outcome.

## Patients and methods

### Patients

After approval from the Medical Ethical Committee, we searched in the surgical procedures and diagnosis database for all patients who were surgically treated for a new diagnosis of SCFE between January 1, 1980 and December 31, 2002 in four tertiary pediatric referral centers. Inclusion criteria were new diagnosis of SCFE treated with in situ pinning. The minimum follow-up was 10 years. The exclusion criteria were endocrine conditions, particularly kidney diseases, and unknown severity of the slip at primary presentation and treatment.

The medical records were retrospectively reviewed for demographic data, date of surgery, surgical method, medical history at the time of diagnosis, outcome of surgery, and the need for further procedures. Southwick angles were measured on the primary frog-leg lateral radiographs at the time of presentation and on the first postoperative X-rays, and the severity of the slip was graded as mild, moderate or severe, as previously described by Southwick [9]. In cases where the primary radiographs were not available, we quoted the description of angle or severity from the radiology reports or the medical records.

**Fig. 1:**
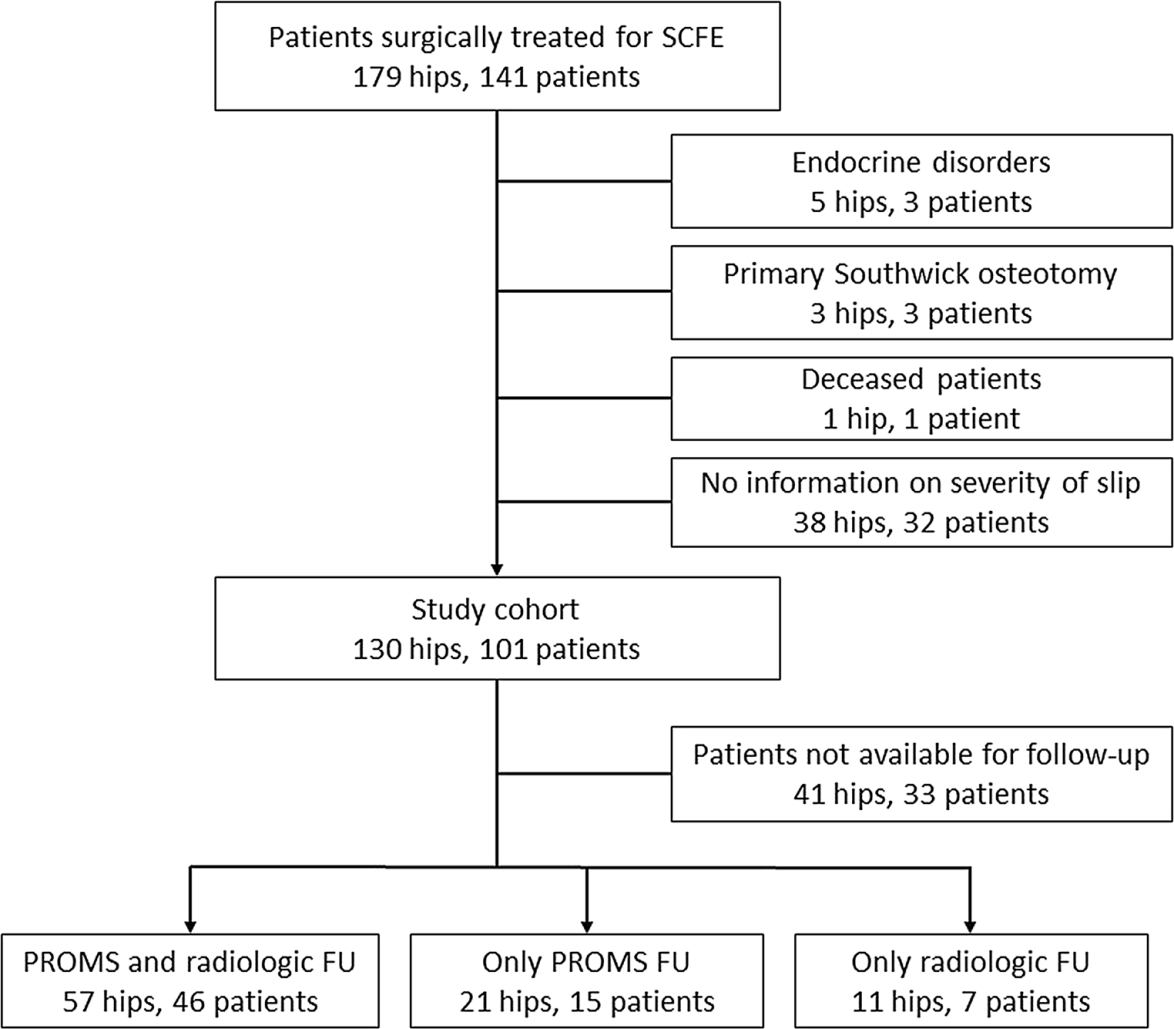
Tree diagram of exclusion and inclusion of patients

Figure 1 shows a tree diagram of excluded and included patients. One hundred and forty-one patients (179 hips) were diagnosed and surgically treated for idiopathic SCFE at the four hospitals. One patient had died. Three patients were primarily treated with Southwick osteotomy. Three patients with five SCFEs had endocrine disorders (all three had kidney failure). In 32 patients no information could be found on severity of the slip at the time of presentation. This left 101 patients (130 hips) for our study cohort. There were 55 boys and 46 girls. Mean age at diagnosis was 14.2 years (range 10.0–20.1) for the boys and 11.7 years (range 8.1–13.6) for the girls. Patients were treated with multiple pins or with a single screw. Patients who were treated with multiple pins were mostly treated before 1994, and patients who were treated with a single screw were mostly treated after 1994. Postoperative management was not the same for all patients, but most patients had partial weight-bearing or no weight-bearing with two crutches for at least 6 weeks. Follow-up for all patients consisted of radiography of the hip in AP and Lauenstein position until skeletal maturity. Full weight-bearing was generally allowed after 6 weeks in the absence of hip pain. All patients were invited by letter to come to the outpatient clinic for physical examination and radiography, and patient-reported outcome measurement (PROM) forms were sent by mail. Several attempts were made by letter and by telephone to contact all of the patients. All patients who participated completed written informed consent. PROMs were available for 61 patients (78 hips) with a mean follow up of 18.4 years (range 11.2–30.2 years) after in situ pinning. Final follow-up X-rays were available for 53 patients (68 hips).

### Radiological evaluation

Standard AP and frog-leg lateral radiographs had been obtained at the time of diagnosis and shortly after surgery. Pre- and postoperative radiographs were available for 72 hips. The slipping angle or Southwick angle (SA) was measured by two orthopedic surgeons independently. Whenever the measurements disagreed by more than 5°, the angles were measured again. The SA is defined as the difference between the affected side and the normal side in the angle between the line connecting the corners of the femoral epiphysis and a line perpendicular to the longitudinal axis of the femoral shaft on the frog-leg lateral view. When both sides are affected, 12° is subtracted from the angle measured. The slip is then classified as mild when less than 30°, moderate when 30°–50°, and severe when more than 50° slipping angle [9]. Besides the SA angle, the postoperative radiographs were reviewed for adequacy of positioning of the pins/screws, number of pins or screws, and perforation of the joint. At follow-up, AP pelvis and frog-leg lateral or normal lateral X-rays were made. These were scored by two orthopedic surgeons for signs of osteoarthritis using the Kellgren–Lawrence classification [14].

### Patient-reported outcome measurements and clinical results

Patient-reported outcome measurements (PROMs) were available for 78 hips. Hip function and impairment was evaluated using the HOOS, EQ5D, and SF36 scores. The hip dysfunction osteoarthritis outcome score (HOOS) is a validated scoring instrument with five subscores on pain, mechanical symptoms, difficulties in activities of daily living, sports, and quality of life [15]. The HOOS is completed for the affected hip. When the patients had bilateral SCFE they filled in one form for the left and another one for the right hip. As a measure of health-related quality of life, we used EQ5D (EuroQol) [16]. The EQ5D is a standardized instrument for measuring health outcome and has five dimensions (mobility, personal hygiene, usual activities, pain/discomfort, and anxiety/depression). The EQ5D visual analogue scale (VAS) ranges from 0 (worst possible health) to 100 (best possible health). The SF36 is a multi-purpose, short-form health survey with 36 questions. It yields an 8-scale profile of functional health and well-being scores as well as psychometrically-based physical and mental health summary measures and a preference-based health utility index. It is a generic measure, as opposed to one that targets a specific age, disease, or treatment group [17].

### Statistical analysis

Baseline patient demographics and clinical data are described for the entire cohort, and were compared between grades of slip. SPSS (IBM SPSS Statistics 21) was used for the statistical analysis. Independent samples *t* test, chi-squared test and one-way ANOVA were used to find differences between groups. Kaplan–Meier survival analysis was done to study the time before presence of osteoarthritis in patients with mild or moderate, or severe slips. Log-rank testing was performed to compare the curves and Cox regression was used to analyze prognostic factors. *p*-values of less than 0.05 were considered significant.

## Results

Eighty-nine hips in 68 patients were available for follow-up (PROMs and/or radiological follow-up).

Sixty-one of the patients included completed the PROMs, and 53 came to the outpatient clinic for radiological follow-up (Fig. 1). To investigate whether the patients who completed the PROMs and/or visited the outpatient clinic were a representative sample of the total group of included patients, we compared gender, grade of slip, age at surgery, and length of follow-up between the groups who participated in both the outcome measurements, and the group that did not. Patients who completed the PROMs had a longer follow-up time after surgery (18.4 ± 5.5 versus 16.6 ± 4.3 years, *p* = 0.05) and a more severe SCFE, as measured by Southwick angle (29.2° ± 18° versus 21.0° ± 16°, *p* = 0.02). There were no differences between the patients who came to the outpatient clinic for radiological follow-up and the patients that did not. In the contralateral normal hips, mean Southwick angle was 10.6°, with a standard deviation of 6.1° (range 1°–23°).

**Fig. 2:**
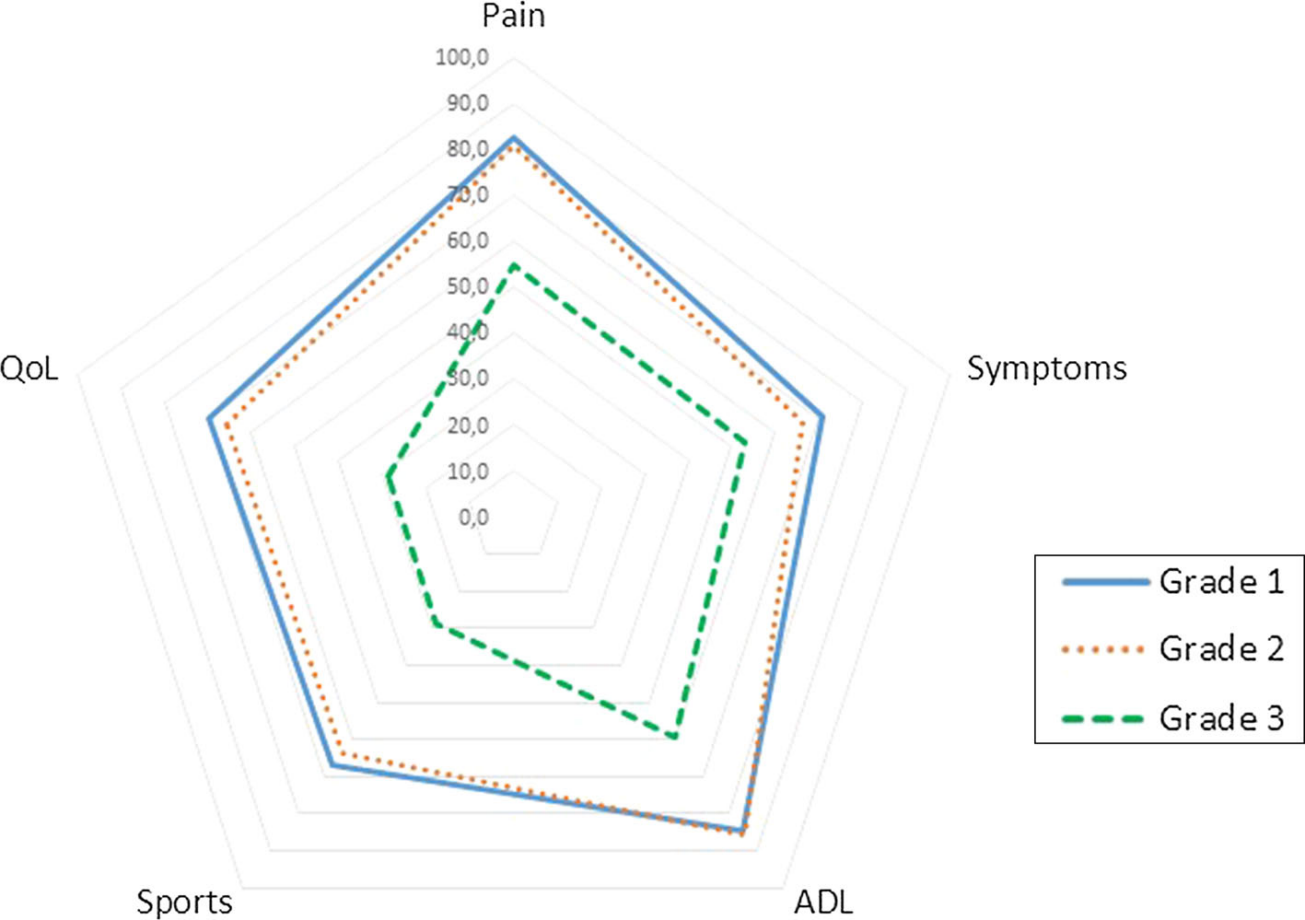
Radar graph of HOOS outcome for grade 1, 2, and 3 slips (according to Southwick angles). HOOS scores are shown for pain, symptoms, activities of daily living, sports, and quality of life

**Fig. 3:**
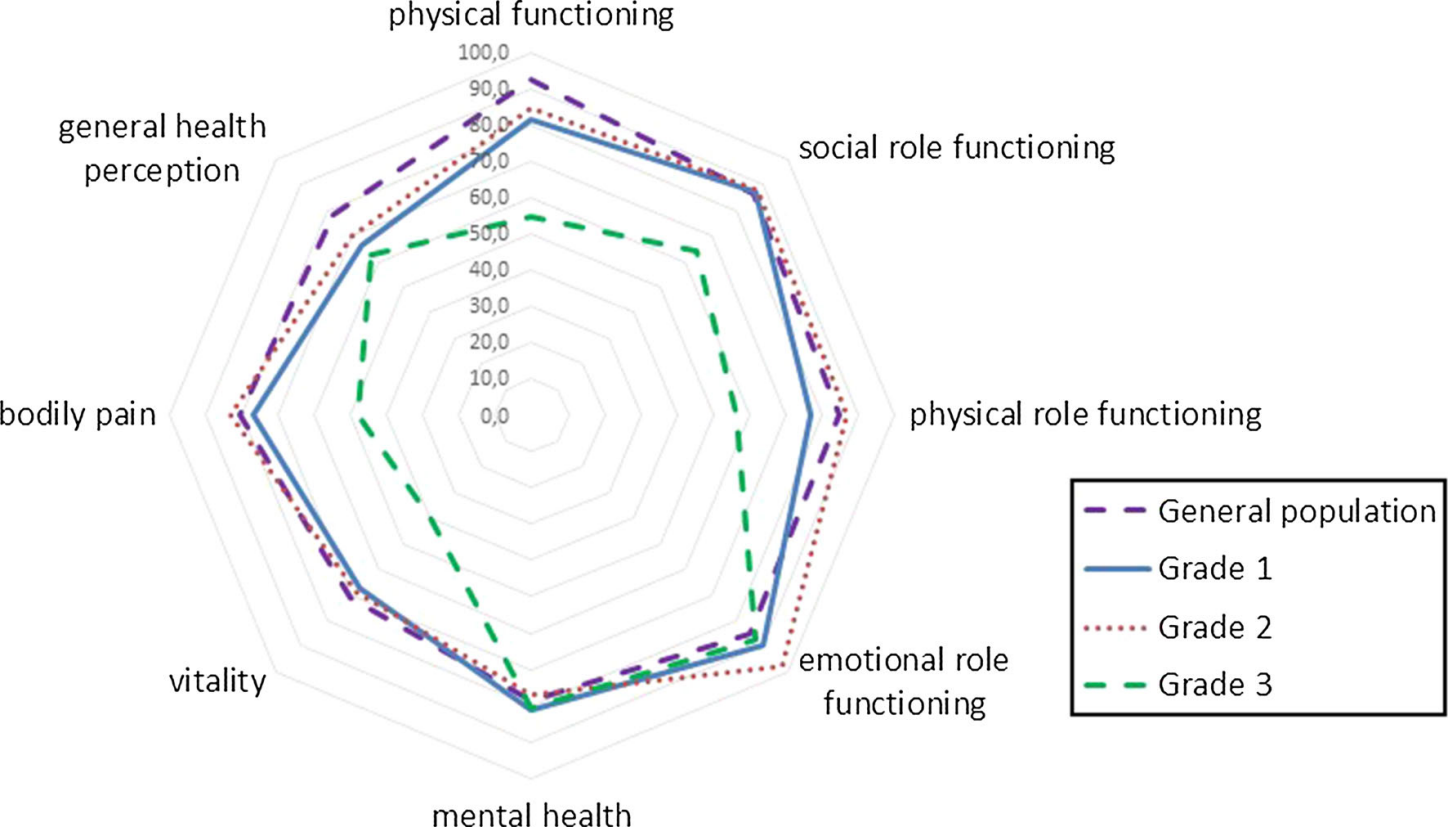
Radar graph of SF36 outcomes based on slip grade according to Southwick angle. The *blue continuous line* shows norm-based data from a general population stratified for age and gender

To determine the influence of severity of slip on functional outcome, we measured HOOS for every involved hip, and the SF36 and EQ5D for every patient. Table 1 shows the EQ5D for all patients who completed the PROMs, divided by grade of slip, as well as some demographic and clinical characteristics. There were no differences observed in age, gender, side of involved hip, number of pins, and follow-up period. Patients with a severe slip had a lower EQ5D than patients with mild or moderate slips, but the EQ5D–VAS showed no significant differences. Figure 2 and Table 2 show HOOS scores for different grades of slip. There were no differences in HOOS sub-scores between hips with mild or moderate slip. Hips with severe slips showed lower scores on all sub-scores except for the sub-score “Symptoms”. Figure 3 and Table 3 show SF36 sub-scores per grade of slip, and comparison with normative values matched for age and gender. For the SF36 there were no differences in scores between mild and moderate slips. However, patients with a severe slip scored lower on physical functioning, social role functioning, vitality, and bodily pain.

**Table 1:** Demographic and clinical data for patients who completed the PROMs, as well as EQ5D reports for these patients

	Grade of slip			Total	*p*-value
	1	2	3		
Gender					
Female	25 (53 %)	10 (43 %)	3 (37 %)	38 (49 %)	0.95*
Male	22 (47 %)	13 (57 %)	5 (63 %)	40 (51 %)	
Side					
Left	26 (55 %)	13 (57 %)	5 63 %)	44 (56 %)	0.93*
Right	21 (45 %)	10 (43 %)	3 (37 %)	34 (44 %)	
Number of pins					
1	22 (48 %)	16 (69 %)	3 (38 %)	41 (53 %)	0.42*
2	15 (33 %)	5 (22 %)	2 (25 %)	22 (29 %)	
3 or more	9 (19 %)	2 (19 %)	3 (37 %)	14 (18 %)	
Age at time of surgery, years (mean ± SD)	12.7 ± 2.2	13.4 ± 2.8	13.4 ± 2.8	13.1 ± 2.4	0.07^#^
Follow up in years (mean ± SD)	18.4 ± 5.7	18.1 ± 5.2	19.4 ± 5.3	18.4 ± 5.5	0.85^#^
EQ5D score (mean ± SD)	0.85 ± 0.23	0.92 ± 0.12	0.48 ± 0.29	0.83 ± 0.23	0.00^#^
EQ5D VAS (mean ± SD)	81 ± 17	77 ± 11	72 ± 14	79 ± 16	0.38^#^

* Chi-squared test

^#^ *t*-Test for independent samples

*SD* standard deviation

**Table 2:** HOOS outcome for grade 1, 2, and 3 slips (according to Southwick angles)

HOOS	Grade of slip				*p* value
	1	2	3	Total	
Pain (mean ± SD)	82.6 ± 21.5	81.1 ± 22.3	54.7 ± 22.3	79.3 ± 22.9	0.01^#^
Symptoms (mean ± SD)	70.6 ± 24.3	66.1 ± 23.5	53.1 ± 23.0	67.5 ± 24.1	0.16^#^
ADL (mean ± SD)	84.8 ± 20.4	85.6 ± 19.3	59.6 ± 21.5	82.5 ± 21.2	0.01^#^
Sport (mean ± SD)	66.8 ± 31.5	63.6 ± 32.8	28.9 ± 25.0	61.9 ± 32.7	0.01^#^
QoL (mean ± SD)	69.6 ± 29.1	65.9 ± 25.9	28.9 ± 19.7	64.3 ± 29.5	0.01^#^
Total (mean ± SD)	74.4 ± 23.3	72.4 ± 22.7	45.1 ± 20.6	71.0 ± 24.2	0.01^#^

HOOS scores are shown for pain, symptoms, activities of daily living, sports, and quality of life. Mean, standard deviations, and *p*-values are shown

^#^ ANOVA

*SD* standard deviation

**Table 3:** SF36 outcome for grade 1, 2, and 3 slips and total group

	Grade of slip				*p* value
	1	2	3	Total	
SF36 physical functioning (mean ± SD)	82 ± 23	85 ± 19	55 ± 20	80 ± 16	0.00^#^
SF36 social role functioning (mean ± SD)	88 ± 19	88 ± 22	64 ± 29	85 ± 22	0.01^#^
SF36 physical role functioning (mean ± SD)	79 ± 33	88 ± 29	56 ± 42	79 ± 33	0.08^#^
SF36 emotional role functioning (mean ± SD)	90 ± 24	98 ± 8	88 ± 25	92 ± 21	0.31^#^
SF36 mental health (mean ± SD)	81 ± 13	78 ± 12	88 ± 25	80 ± 13	0.61^#^
SF36 vitality (mean ± SD)	67 ± 16	69 ± 15	40 ± 22	64 ± 18	0.00^#^
SF36 bodily pain (mean ± SD)	78 ± 26	84 ± 19	48 ± 26	76 ± 26	0.00^#^
SF36 general health perception (mean ± SD)	67 ± 21	70 ± 18	63 ± 18	67 ± 20	0.70^#^

Mean, standard deviations, and *p* values are shown

^#^ ANOVA

*SD* standard deviation

**Fig. 4:**
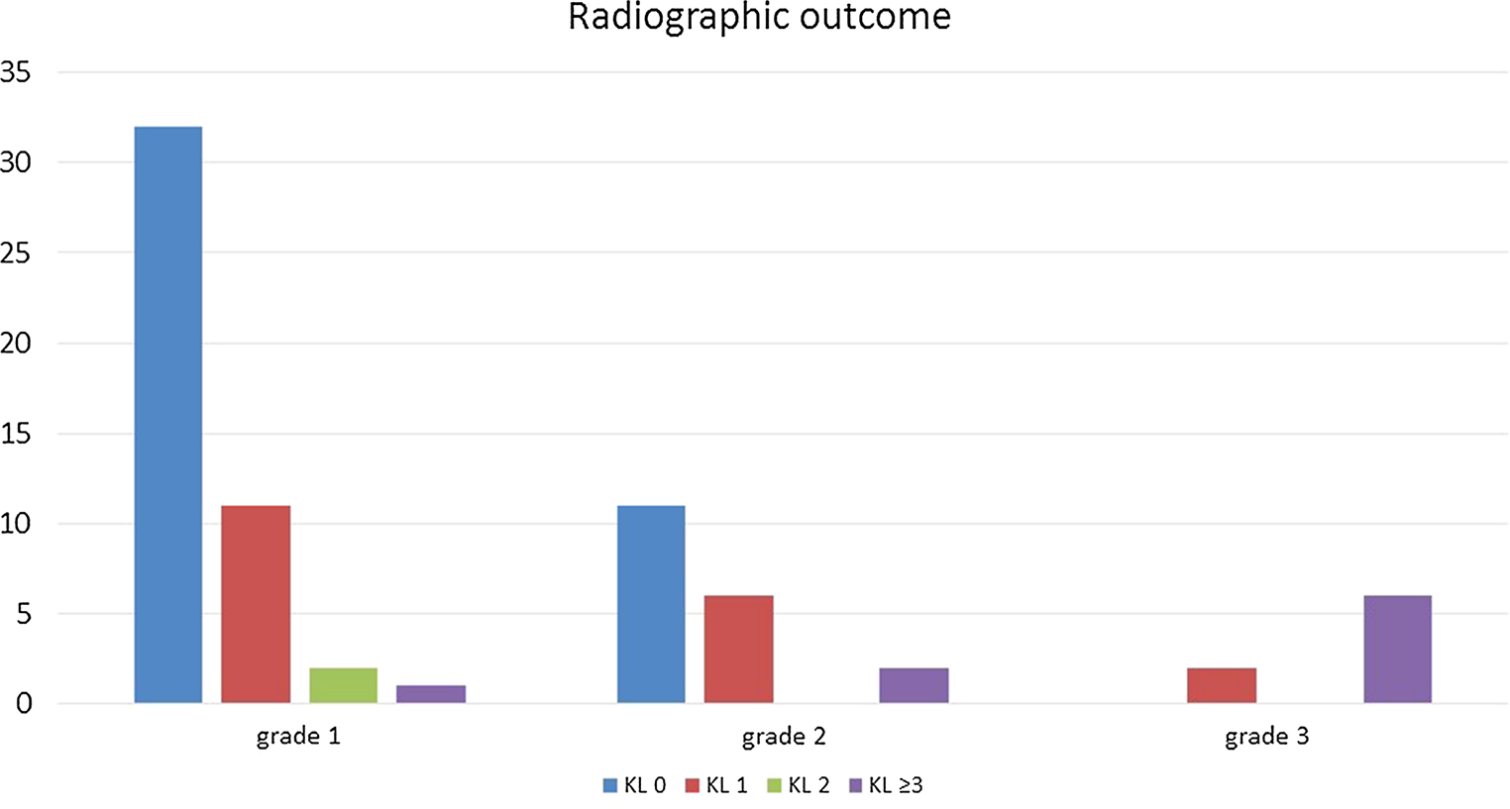
Radiographic outcome (osteoarthritis) based on slip grade. The group KL ≥3 contains hips with osteoarthritis grade 3 and 4 according to Kellgren and Lawrence, as well as hips that were already replaced with a total hip prosthesis

Fifty-three patients with 68 slipped femoral heads were evaluated at the outpatient clinic with radiological follow-up. The five patients who had undergone total hip replacement were not asked to come to the outpatient clinic for radiological follow-up as these X-rays were not contributing to the outcome. X-rays made before the total hip replacement showed grade 3 and 4 osteoarthritis (in two patients due to osteonecrosis) in all five patients. Figure 4 shows distribution of radiological outcome per slip grade. Four categories of osteoarthritis were considered. The first three categories were grades 0, 1, and 2 according to the Kellgren and Lawrence scale. In the last group, patients with severe osteoarthritis were categorized, namely, Kellgren and Lawrence scale 3 and 4, and patients who already had total hip replacement. In the hips with mild slip, one of 46 patients (2 %) showed severe osteoarthritis. In hips with moderate slip, 11 % of patients were classified as severe osteoarthritis, and in the patients with severe slip, 75 % had severe degenerative changes on radiographs.

At the end of follow-up five patients had total hip replacement, and four patients had undergone Southwick osteotomy, of which one patient had subsequent hip arthrodesis. In one patient with grade 3 slip, ongoing complaints and severe slip resulted in the decision to perform a Dunn procedure at age 18. Two years later, in 1993, a total hip replacement was performed due to osteonecrosis. In 1996 and 1999, revision total hip arthroplasties were done because of loosening of the hip prosthesis. Another patient with grade 3 slip had a total hip prosthesis 12 years after pinning, due to serious malformation of the femoral head. One patient had total hip replacement 21 years after grade 3 slip. In a patient with grade 2 slip, osteonecrosis occurred after pinning and a total hip prosthesis was placed 16 years after the initial surgery. In one patient with grade 1 slip, total hip arthroplasty was done 13 years after pinning, due to osteoarthritis with pain and limitations in range of motion. Four patients underwent Southwick osteotomy secondary to pinning of the slip. One of these patients had grade 2 slipping, the three other patients had grade 3. The patient with grade 2 slipping had a Southwick osteotomy 10 years after the initial pinning. The patients with a grade 3 slip underwent Southwick osteotomy at 1, 3, and 6 years after the initial pinning. In the patient who was operated 3 years after pinning, osteonecrosis was suspected. Two years after the osteotomy there was a complete destruction of the femoral head and consequently a hip arthrodesis was performed. Of the three patients who underwent Southwick osteotomy without the subsequent arthrodesis, two patients had osteoarthritis grade 1 according to the Kellgren and Lawrence scale at 2 and 6 years follow-up after the osteotomy. One patient had grade 3 osteoarthritis 15 years after the osteotomy. Patients with severe slips had a significantly worse survival than patients with mild or moderate slips (*p* = 0.01). A multivariate Cox regression model entering severity of slip, age, and gender resulted in a hazard ratio for mild to severe slips of 0.1 (0.01–0.6). Hazard ratios were insignificant for age 1.8 (0.2–14) and gender 0.9 (0.6–1.2) in this analysis.

In seven patients with acute on chronic slips, inadvertent reposition was seen after surgery. One of these seven patients, who had a change in SA from 56° to 28°, had grade 3 osteoarthritis at 16 years follow-up and one patient who had a change in angle from 60° to 40° had undergone a Southwick osteotomy 6 years after primary surgery. The other patients had no subsequent surgeries or complications, and, in general, had better outcomes than patients with chronic slips of the same grade (Table 4). Three patients had additional surgery because the pins were out of the epiphysis at follow-up. In one of these patients a further slip had occurred. All three had good outcome and no signs of osteoarthritis at follow-up.

**Table 4:** Southwick angles, HOOS scores, radiological outcome, complications, and long-term outcomes of the seven patients with acute on chronic slips

	SA preoperative	SA postoperative	HOOS score	SF36 score	Radiological outcome	Complications and outcomes
1	56°	28°	70.6	71.4	KL 3	Screw removal 8 years after surgery due to complaints
2	60°	40°	46.5	68.5	KL 1	Southwick osteotomy 6 years after primary surgery
3	25°	23°	100	85.4	–	–
4	33°	30°	57.9	–	KL 0	–
5	60°	13°	93.1	51.8	–	–
6	97°	12°	67.7	67.9	–	–
7	60°	25°	–	–	KL 1	–

*SA* Southwick angle, *KL* Kellgren and Lawrence grade

## Discussion

This study investigates the functional and radiological outcome after in situ pinning at a mean follow-up of 18.4 years postoperatively. We aimed to determine whether patients with a more severe slip had a higher risk of worse functional or radiological outcome. We investigated the long-term functional and radiological results after in situ fixation for SCFE, and determined whether hips with more severe slips had a worse outcome than moderate or mild slips. There were no differences between age at surgery, gender, side of slip, follow-up period, or number of pins between groups. Although mild and moderate slips showed no differences in functional and radiological outcome, hips with severe slips scored significantly lower on HOOS and EQ5D, and the physical functioning, social role functioning, vitality, and bodily pain sub-scales of the SF36. The sub-scales of the SF36 with no differences between slip grades (general health perception, emotional role functioning, and mental health) are probably less influenced by functional problems and pain. In the total group of 65 mild or moderate slips two patients had total hip replacement, and one patient had grade 3 osteoarthritis according to Kellgren and Lawrence (total of 5 %). In the group of eight severe slips one patient had undergone a hip arthrodesis, three patients had total hip replacement, and two patients had grade 3 osteoarthritis of the hip at radiological follow-up. Thus, 75 % of patients with severe slips had early-onset osteoarthritis of the hip. Patients with acute on chronic slips in general had better outcomes than patients with chronic slips of the same grade. This can be explained by the fact that after the inadvertent reposition the slip can be improved to a milder grade. When the reposition itself does not cause additional complications, the reposition will be beneficial.

There are a few limitations to our study. In 37 of 138 patients treated for SCFE between 1980 and 2002 in four hospitals, no information could be found on severity of the slip, and these patients therefore had to be excluded, leaving 101 potential patients for the study. Despite extended efforts to reach and motivate all patients to complete the PROMs and/or visit the outpatient clinic for radiological follow-up, only 68 patients (67 %) committed to completing the PROMs or making the follow-up X-rays. Gender and age at surgery were equal in the patients who entered the study and the patients who did not. Patients who completed the PROMs had a longer follow-up and a higher Southwick angle than patients who did not. This last feature can be explained by the fact that some of the patients we called to participate in the study did not want to do so because they had no functional problems of the hip. Patients with complaints are probably more motivated to participate.

In the past, a few studies have focused on long-term follow-up of SCFE. Boyer et al. described, in 1981, a group of 121 patients with SCFE and concluded that mild and moderate slips could be safely pinned in situ, based on clinical and radiological follow-up [18]. The drawback of this study by Boyer is that all patients were operated on before 1952, and the techniques that were used at that time are not easily comparable with the current techniques. In 1991 Carney et al. again described the Boyer group, but now with 10 more years follow-up [19]. Hansson et al. studied 43 patients with 59 slipped capital femoral epiphyses, all treated with in situ pinning [20]. They used Harris Hip Score (HHS) and radiological examination to define clinical outcome. The mean HHS was 97 in patients with mild slip and 74 in patients with severe slips, but the differences were not statistically significant (*p* = 0.13). Their recommendation was to use in situ pinning for mild slips, and probably also for moderate slips, until there is more evidence [20]. In 2012 Larson et al. studied long-term functional and radiological follow-up in 84 patients [21]. They concluded that, unexpectedly, although high-grade slips were associated with poorer outcome scores, mild slips also frequently became symptomatic [21]. Lately, Escott et al. have reported long-term follow-up of 64 patients with SCFE. They included patients with SCFE treated with in situ fixation without reduction of the slip. Patients with comorbidities that predisposed to SCFE and with missing data were excluded. They documented PROMs and determined that there was no association between higher slip angle and poorer health outcome [22]. One of the explanations for the lack of differences given by Escott et al. is that the patients may have altered their activities, masking the more subtle functional deficits or pain associated with the deformity. We did find worse outcomes in patients with severe slips. However, the number of patients with severe slips is low in both studies, making the results less powerful.

This study underlines the good short- and long-term results of in situ pinning in mild to moderate slips. We conclude that severe slips have a significantly worse clinical outcome after in situ pinning, and open reduction and internal fixation may be considered in these hips. Hips with mild and moderate slipped capital femoral epiphysis show excellent long-term functional and radiological outcome at a mean follow-up of 18 years, and for these hips there seems to be no indication for open procedures.
